# Proapoptotic and Antiproliferative Effects of the Desert Truffle *Terfezia boudieri* on Colon Cancer Cell Lines

**DOI:** 10.1155/2023/1693332

**Published:** 2023-04-07

**Authors:** Katia Sawaya, Sonia Abou Najem, Ghada Khawaja, Mahmoud Khalil

**Affiliations:** ^1^Department of Biological Sciences, Faculty of Science, Beirut Arab University, Beirut, Lebanon; ^2^Health Sciences Division, Abu Dhabi Women's College, Higher Colleges of Technology, Abu Dhabi, UAE; ^3^Molecular Biology Unit, Department of Zoology, Faculty of Science, Alexandria University, Alexandria, Egypt

## Abstract

**Background:**

Colon cancer is the second leading cause of cancer-related mortality, and ranks third among cancers in terms of prevalence. Despite advances in early detection and treatment with chemotherapy and surgery, colon cancer continues to be associated with high recurrence rates, thereby resulting in a heavy disease burden. Moreover, the effectiveness of currently available treatment modalities is limited by the occurrence of toxic side effects. Hence, there is an urgent need to develop alternative treatments. Extracts from the black desert truffle *Terfezia boudieri* (*T*. *boudieri*) have shown promising anticancer properties. However, the cellular mechanisms underlying this activity remain poorly understood.

**Methods:**

In this study, the colon cancer cell lines HCT-116 and Caco-2 were treated with either water or ethanolic extract of *T*. *boudieri*. Cell viability and the half-maximal inhibitory concentration were determined using MTT assays. Then, the activity of the more potent water extract was further verified using crystal violet assays, and its role in inhibiting colony formation and wound healing was investigated. Protein levels of p53, B-cell lymphoma 2 (Bcl-2), Bcl-2-associated X (Bax), cyclin D1 (CCND1), and c-Myc were measured in cells treated with different doses of the water extract.

**Results:**

Treatment with the water extract of *T*. *boudieri* reduced the capacity of cells for wound healing and colony formation in a dose-dependent manner. The Bax/Bcl-2 ratio and p53 expression were elevated in both cell lines. In contrast, the levels of cyclin D1 and c-Myc were suppressed.

**Conclusion:**

*T*. *boudieri* water extract exerted a cytotoxic effect on colon cancer cells, and blocked colony formation and wound healing potentially through inhibition of proliferation. Mechanistically, these effects are attributed to influence the mitochondrial pathway of apoptosis, proteins involved in cellular proliferation, and the cell cycle.

## 1. Introduction

Colorectal cancer is the second leading cause of cancer-related death worldwide and the third most common type of cancer [1]. The incidence of colon cancer continues to increase worldwide due to unhealthy lifestyles and food habits [2, 3]. Tumor cells possess innate tolerance or acquire de novo tolerance to presently available chemotherapy and radiotherapy techniques [4]. This ability results in high postsurgical recurrence rates, which are associated with the poor 5-year survival rates.

Similar to other types of cancer, colon cancer develops due to mutations arising in genes responsible for DNA repair and tumor suppression, such as p53 [5] (also termed as guardian of the genome), as well as several oncogenes. In turn, these mutations dysregulate important cell signaling pathways involved in cell proliferation, cell cycle progression, and apoptosis [6, 7]. It has been shown that c-Myc increases the expression of proteins involved in cellular proliferation. Also, cyclin D1 induces transformation and chemoresistance. Therefore, c-Myc and cyclin D1 have attracted considerable attention as potential targets for the treatment of cancer [8, 9].

As key regulators of cellular apoptosis, proteins belonging to the B-cell lymphoma 2 (Bcl-2) family may also be important targets in this setting. For example, Bcl-2-associated X (Bax) promotes this type of regulated cell death, while Bcl-2 prevents apoptosis by interfering with the activity of Bax to induce mitochondrial membrane permeability [10]. Numerous studies have proposed that the Bax/Bcl-2 ratio can serve as an important prognostic marker of clinical outcomes for patients with many types of cancers, including colon cancer [11–15].

The need for treatment alternatives that may improve outcomes for patients with cancer continues to fuel the search for natural medicinal sources, particularly among plants and mushrooms [16].

*Terfezia boudieri* (*T*. *boudieri*) is a desert truffle that grows around the Mediterranean basin. Documentation pertaining to the usage of this desert truffle as food and medicine dates back to pre-Islamic times [17]. Many studies have analyzed the chemical composition of *T*. *boudieri*, demonstrating its high nutritional value. It is mainly composed of carbohydrates (∼60%), followed by proteins and fatty acids (particularly linoleic acid), as well as an amplitude of minerals (e.g., calcium, potassium, phosphorus, and iron). In addition, the truffle contains carotenoids, anthocyanins, flavonoids, and phenolics which confer a potent antioxidant activity [18–20].

According to Al-Obaydi et al., the methanolic, water, n-hexane, ethyl, and acetate extracts of *T*. *boudieri* contain carbohydrates, terpenoids, and phytosterols. However, tannins, alkaloids, and flavonoids were only found in the methanolic and water extracts, which also contained the highest amounts of gallic acid; notably, the highest yield was obtained from the water extract [21]. Components of *T*. *boudieri*, such as phytosterols [22, 23], terpenoids, [24] flavonoids [25, 26], and tannins, possess antioxidant and antibacterial properties [27]. Thus, it is expected that *T*. *boudieri* extracts would also possess such properties. Indeed, the antioxidant and antibacterial activities of *T*. *boudieri* have been demonstrated [28]. Furthermore, extracts of this truffle have shown an immunomodulatory capacity. Following treatment with the aforementioned extracts, lymphocytes displayed an increased rate of proliferation and secreted significantly higher levels of interferon-*γ* (IFN-*γ*) and interleukin-2 (IL-2). Similarly, macrophages responded to the exposure to *T*. *boudieri* extracts, and the highest phagocytic activity was observed in those treated with the water extract. *T*. *boudieri* extracts are also able to block angiogenesis by inhibiting the expression of vascular endothelial growth factor (VEGF) [21].

Through the usage of cell viability assays, several studies involving various cancer cell lines (e.g., HEPG2, HELA, Caco-2, HCT116, HT29, MCF-7, PC3, U-87 MG, T47D, and MDA-MB231) have demonstrated the anticancer potential of *T*. *boudieri* extracts [21, 29]. However, the mechanisms by which *T*. *boudieri* exerts its anticancer effects remain poorly investigated.

## 2. Materials and Methods

### 2.1. Preparation of *T*. *boudieri* Crude Extracts

*T.boudieri* ascomycetes were purchased from a local market in Lebanon. Subsequently, they were washed by scrubbing, cut into thin slices, air-dried, and ground into fine powder. The dried powder was homogenized in a ratio of 1 : 4 (weight/volume) in either distilled water or 90% ethanol. After 24 h of shaking at room temperature, the homogenates were filtered through Whatman filter paper and lyophilized into a soft paste. The water extract was prepared for treatment by weighing and dissolving a stock solution of 20 mg/ml, and filtered using a 33-mm syringe filter (Sigma-Aldrich, Taufkirchen, Germany). The stock was diluted to various concentrations using complete Dulbecco's Modified Eagle Medium (DMEM) (Sigma-Aldrich). The ethanolic extract stock solution was first dissolved in dimethyl sulfoxide (DMSO) (Sigma-Aldrich), diluted with DMEM, and filtered with a 33-mm syringe filter (Sigma-Aldrich). The percentage of DMSO in the highest treatment dose was 3%.

### 2.2. Maintenance of Colon Cancer Cell Lines

HCT-116 and Caco-2 cell lines were provided by the cell culture facility of the Faculty of Science, Alexandria University (Alexandria, Egypt). These cells were originally purchased from the American Type Culture Collection (Manassas, VA, USA). Cells between 8 and 12 passages were used in the experiments. They were cultured in complete medium DMEM (Lonza, Bend, OR, USA) and supplemented with 10% fetal bovine serum (Sigma-Aldrich), 1% L-glutamine (Sigma-Aldrich, St. Louis, MO, USA), and 1% antibiotic-antimycotic (Biowest, Bradenton, FL, USA). Cells were maintained in an incubator at 37°C with 5% CO_2_ and 95% humidity. Routine evaluations for the detection of mycoplasma contamination were conducted. There was no mycoplasma contamination detected in these cell lines.

### 2.3. Cell Viability Assay

The 3-(4,5-dimethylthiazol-2-yl)-2,5-diphenyltetrazolium bromide (MTT) assay was used to determine the cell viability. After reaching 70–80% confluence, the cells were incubated in 96-well plates (density: 3 × 10^4^ cells per well) under standard conditions and exposed to decreasing concentrations of either water or ethanolic extracts (i.e., 10–0.75 mg/ml) for 48 h in triplicates. Following treatment, 5 mg/ml MTT (10 *μ*l) (Thermo Fisher, Waltham, MA, USA) was added to each well, the mixture was filtered through a 33-mm syringe filter (Sigma-Aldrich), and the cells were incubated for 4 h. Thereafter, isopropanol (100 *μ*l) was added to each well, and the cells were incubated overnight. Optical density (OD) was measured at 595 nm using a microplate reader (Multiskan FC, Sigma-Aldrich). Negative controls were included containing either complete DMEM or DMEM with the same concentration of DMSO as that present in the ethanolic extract solutions. Cell viability percentage was calculated using the following formula:

(1) $$\begin{array}{c} Cell\;viability\left(\%\right)=\left[\frac{\left({OD}_{sample}–{OD}_{blank}\right)}{\left({OD}_{control}–{OD}_{blank}\right)}\right]\times 100\text{.} \end{array}$$

### 2.4. Crystal Violet Assay

Cells (density: 3 × 10^4^) were seeded in 96-well microtiter plates and exposed to different concentrations of the water extract for 48 h. Subsequently, the cells were stained with 200 *μ*l of 0.5% crystal violet for 30 min and washed with tap water. The absorbance was measured at 492 nm (Multiskan FC, Sigma-Aldrich).

### 2.5. Cytomorphology

Cells were seeded in 12-well plates and exposed to three different concentrations of water extract (half maximal inhibitory concentration (IC_50_), 1/2 IC_50,_ and 1/4 IC_50_) for 48 h in duplicates. Thereafter, the cells were stained with 200 *μ*l of 0.5% crystal violet for 30 min and washed with tap water. Three random frames of each well were photographed under a microscope (B120-DK AmScope, Irvine, CA, USA) at 40x and 200x magnifications.

### 2.6. Clonogenic Assay

Cells (density: 1 × 10^3^) were seeded in 12-well plates for 24 h. After reaching 60% confluency, they were exposed to various concentrations of the water extract (IC_50_, 1/2 IC_50_, and 1/4 IC_50_) for 48 h in triplicates. This was followed by the aspiration of the control medium and treatment solutions, the addition of fresh complete medium, and further incubation for 2 weeks. The cells were subsequently stained with 0.5% crystal violet for 30 min and washed with tap water.

### 2.7. Wound Healing Assay

Cells were seeded in 12-well plates for 24 h. After the cells reached 60–70% confluence, a uniform scratch was inflicted in each well using a 10-*μ*l pipette tip. Thereafter, the cells were washed with phosphate-buffered saline (PBS) and treated with different concentrations of water extract (IC_50_, 1/2 IC_50_, and 1/4 IC_50_) or complete DMEM (negative control). After 48 h, three random frames of the wounds were photographed using a microscope (B120-DK AmScope, Irvine, CA, USA) at 40x magnification. The wound area was measured using the ImageJ software (National Institutes of Health, Bethesda, MD, USA; https://imagej.nih.gov/ij/) [30].

### 2.8. Cell Cycle Analysis Using Flow Cytometry

Cells were seeded in two 12-well plates for 24 h. After reaching 60% confluence, the cells were treated with complete DMEM or water extract (IC_50_ and 1/2 IC_50_) for 48 h in duplicates. Then, the cells were trypsinized, washed with ice-cold PBS, centrifuged, and fixed with 70% ethanol under vortexing to avoid clumping. The cell suspensions were centrifuged and rewashed with ice-cold PBS. Subsequently, the DNA was stained with propidium iodide (PI) 300 *μ*g/ml of PI/triton X 100 staining solution (1,000 *μ*l of 0.1% triton + 40 *μ*l PI + 20 *μ*l RNAse). Finally, the cells were analyzed using a BD FACSCanto™ II flow cytometer (BD Biosciences, Franklin Lakes, NJ, USA). Data analysis was performed using the BD FACS Diva software (v 9.0; BD Biosciences).

### 2.9. Western Blotting

Cells were incubated in 12-well plates for 24 h. After reaching 60% confluence, the cells were treated with complete DMEM or water extract (IC_50_ and 1/2 IC_50_) in duplicates. After 48 h, the cells were lysed with cold lysis buffer (100 *μ*l/well) (Bio-Rad, Hercules, CA, USA). The Bradford assay was used to determine the concentration of proteins. Proteins (40 *μ*g) were incubated in Laemmli sodium dodecyl sulfate-polyacrylamide gel electrophoresis sample buffer (Bio-Rad) for 5 min at 98°C and separated on 12% polyacrylamide gels covered with 4% stacking gel at 120 V for 2 h in running buffer (Bio-Rad). Subsequently, the proteins were transferred onto nitrocellulose membranes (Invitrogen, Waltham, MA, USA) in Tris-buffered saline transfer buffer at 35 V for 90 min. Then, the membranes were incubated in 5% nonfat milk for 2 h at room temperature. The membranes were then incubated with Bax rabbit monoclonal antibody (1 : 1,000) (Abcam, Fremont, CA, USA), rabbit anti-Bcl-2 antibody (Abcam), anti-p53 polyclonal antibody (Thermo-fisher, USA), mouse polyclonal anti-CCND1 antibody (Invitrogen), mouse polyclonal anti-c-Myc antibody (Invitrogen), or goat polyclonal anti-*β*-actin antibody (1 : 500) (Abcam) for 2 h at room temperature. Thereafter, the membranes were washed with Tris-buffered saline with 0.1% Tween 20 and incubated with infrared (IR) dye-conjugated secondary antibodies donkey anti-goat, goat anti-rabbit, or goat anti-mouse IRDye 800CW or IRDye 680RD (1 : 100,000) (Abcam). Finally, the membranes were scanned using an Odyssey IR image system (LI-COR Biosciences, Lincoln, NE, USA). Band intensity was measured using the ImageJ software (National Institutes of Health) [30] and normalized to that of *β*-actin. Quantification was conducted using the ImageJ software according to the protocol reported by Davarinejad [31].

### 2.10. Statistical Analysis

Data are presented as mean ± standard error of the mean. The GraphPad Prism version 9 for Windows software (GraphPad Software, La Jolla, CA, USA; http://www.graphpad.com) was utilized to perform a one-way analysis of variance (ANOVA) with Dunnett's posttest. Statistical significance among the groups was determined using the one-way ANOVA. A p value <0.0001 denoted statistically significant differences. Nonlinear regression analysis was used to obtain the IC_50_ for the different extracts of *T. boudieri*.

## 3. Results

### 3.1. Water Extract of *T. boudieri* Provided a Higher Yield than the Ethanolic Extract

The percentage yield upon extraction of 250 g of *T. boudieri* and lyophilization was 12.76% and 4.2% for the water and ethanolic extracts, respectively. This finding demonstrated high variability in yield between the extracts.

### 3.2. *T. boudieri* Extracts Diminished the Viability of HCT-116 and Caco-2 Cells

**Figure 1:**
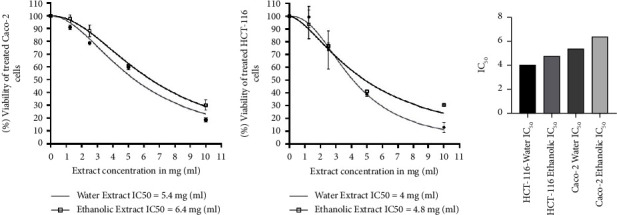
(a) *Terfezia boudieri* water and ethanolic extracts showed a cytotoxic effect on Caco-2 cell line following 48 hs of treatment with different concentrations (10 mg/ml, 5 mg/ml, 2.5 mg/ml, and 1.25 mg/ml). (b) *Terfezia boudieri* water and ethanolic extracts showed a cytotoxic effect on HCT-116 following the same treatment concentrations for 48 hs. (c) A comparison between the different values of the IC_50_ showed a slightly stronger effect for the water extract and more sensitivity of HCT-116 to the treatment.

Exposure of HCT-116 and Caco-2 cells to *T. boudieri* extracts resulted in cancer cell inhibition in a dose-dependent manner; the water extract was slightly more potent than the ethanolic extract. Furthermore, HCT-116 cells were more sensitive than Caco-2 cells to both extracts. The lowest and highest IC_50_ were recorded for HCT-116 cells treated with the water extract (4 mg/ml) and Caco-2 cells treated with the ethanolic extract (6.4 mg/ml), respectively (Figure 1).

### 3.3. Crystal Violet Assay Confirmed the Cytotoxic Effects of *T. boudieri* Water Extract

**Figure 2:**
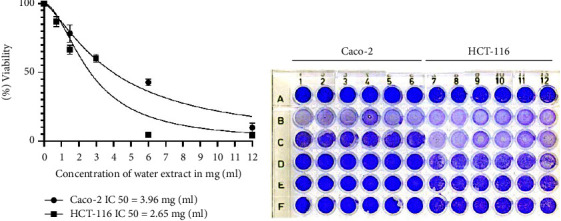
Crystal violet assay showed results similar to those of the cell viability assay using MTT. (a) IC_50_ was lower in HCT-116 treated cells treated with the water extract of *T. boudieri* than that of the Caco-2 cells (2.65 mg/ml and 3.96 mg/ml, respectively). (b) 5% crystal violet dye allowed us to observe the effect of the treatment on the plate with a naked eye. Row A is the negative control, Row B was treated with the highest concentration of water extract (12 mg/ml), and Row F with the lowest (1.25 mg/ml) with the respective dilutions in between, going down from highest to lowest. Columns 1–6 were seeded with Caco-2 cells, whereas columns 7–12 contained HCT-116 cells (3 × 10^4^ cells/well).

Exposure of HCT-116 and Caco-2 cells to *T. boudieri* water extract for 48 h resulted in a dose-dependent cytotoxic effect. HCT-116 cells exhibited higher sensitivity to treatment compared with Caco-2 cells (Figure 2).

### 3.4. Cytomorphological Analysis Revealed Signs of Apoptosis in Treated Cells

**Figure 3:**
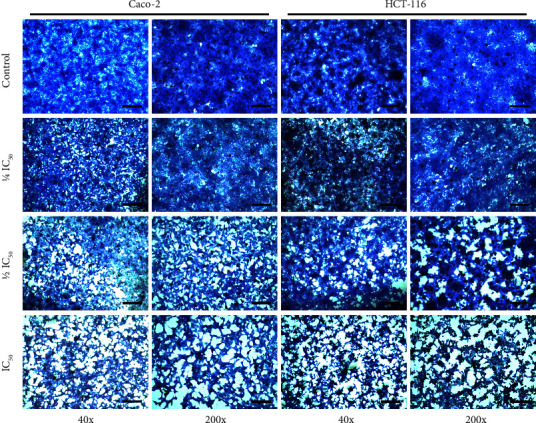
Cytomorphological changes in treated cells showed characteristics of apoptosis in both Caco-2 and HCT-116. Cells were captured at 40x (scale bar 500 *μ*m) and 200x (scale bare 100 *μ*m) magnifications. The changes were dose-dependent. The controls showed a highly confluent and dense monolayer of cells that are more densely stained, this density decreased with increased treatment concentration, and the cells became shrunken and darkly stained as most evident in the wells where the cells were treated with their respective IC_50_ concentrations of the water extract.

Cells exposed to different concentrations of *T. boudieri* water extract showed diminished proliferation and displayed morphological changes consistent with apoptosis (e.g., shrinkage, more intense staining, and dark condensed nuclei) compared with the negative control cells which proliferated into a dense monolayer. The magnitude of changes in treated cells appeared to be dose-dependent (Figure 3).

### 3.5. Treated HCT-116 and Caco-2 Cells Showed a Significantly Decreased Capacity for Colony Formation

**Figure 4:**
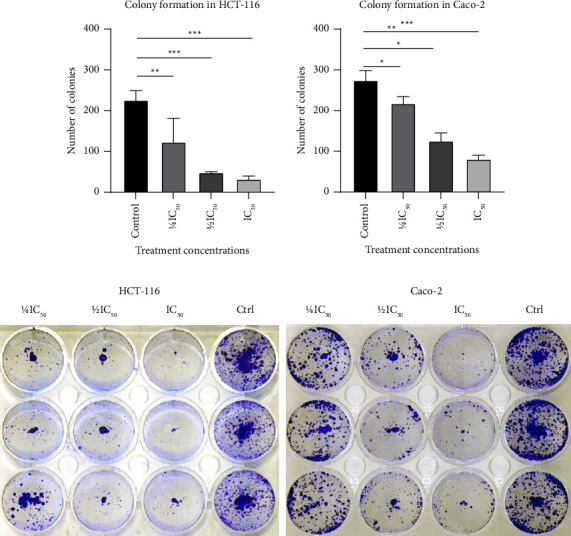
(a) The clonogenic assay showed that *T. boudieri* water extract significantly inhibits colony formation in Caco-2 and HCT-116 cell lines in a dose-dependent manner. Wells containing 1 × 10^3^ cells treated in triplicates showed the most significant inhibition with IC_50_ (4 mg/ml for HCT-116 and 5 mg/ml for Caco-2). Colony inhibition was more prominent in the HCT-116 cell line. (b) Colonies dyed with 0.5% crystal violet are shown to be higher in density in the negative control wells, followed by the wells treated with the lowest concentration of the water extract 1/4 IC_50,_ 1/2 IC_50,_ and IC_50_.

The *T. boudieri* water extract significantly inhibited colony formation in a dose-dependent manner in both cell lines compared with the negative control. The inhibition of colony formation was greater in HCT-116 than in Caco-2 cells, confirming the higher sensitivity of the former cell line to the extract (Figure 4).

### 3.6. *T. boudieri* Water Extract Inhibited Wound Healing in a Dose-Dependent Manner in Treated Caco-2 and HCT-116 Cells

**Figure 5:**
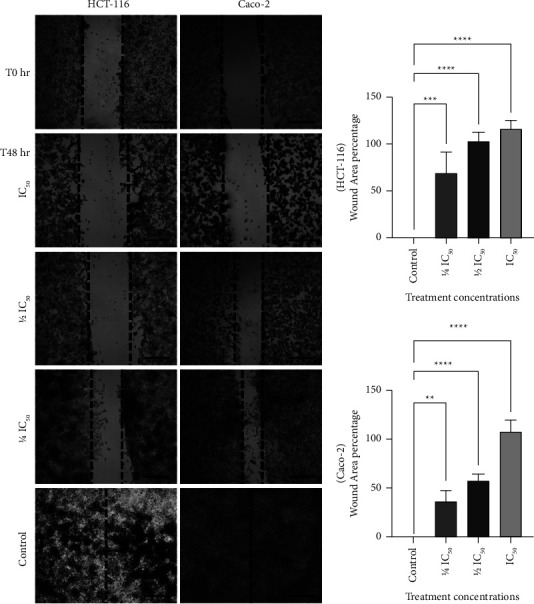
Wound healing capacity diminished significantly in treated cells, especially in HCT-116. (a) There was less migration of the cells toward the center of the wound in a dose-dependent manner. At higher doses of treatment with the water extract of *T. boudieri* (IC_50_) cells displayed apoptotic features and the wound increased in size compared to the time of wound infliction. (b) While the wound completely healed in both negative controls of HCT-116 and Caco-2, the wound was still visible in the wells treated with the lowest concentration of the water extract (1/4 IC_50_). Wound area percentages tended to be greater overall in HCT-116 cells. Wound area was measured using ImageJ (U.S. National Institutes of Health, Bethesda, Maryland, USA, https://imagej.nih.gov/ij/), and wound area percentage was measured according to the formula: % wound area = (AT48/AT0) × 100%, where AT48 hr is the area at 48 hs following treatment and AT0 hrs is the area at the time right after wound induction. Images were captured at 40x magnification scale bar = 500 *μ*m.

The results of the wound healing assay further revealed a dose-dependent inhibitory effect on the proliferation of treated cells. Following treatment, the cells also exhibited a diminished capacity for migration, as well as morphological changes characteristic of apoptosis. At 48 h after treatment with the respective IC_50_, the wound area had increased for both cell lines compared with that recorded at T0 (i.e., the time at which the wound was inflicted on the cellular monolayers). HCT-116 demonstrated a reduction in their capacity for wound healing compared with Caco-2 (Figure 5). These results supported the findings of the previous experiments.

### 3.7. Cell Cycle Analysis Detected Considerable DNA Fragmentation in Treated Cancer Cells

**Figure 6:**
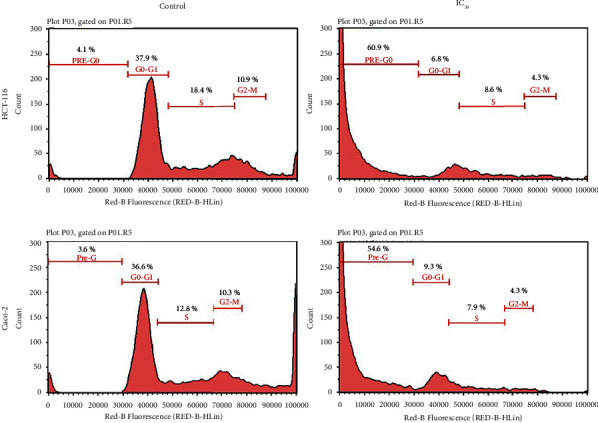
Cell cycle analysis by flow cytometry after 48 hr of treatment detected considerable DNA fragmentation. There was a shift in the peak from G_0_ to G_1_ in the HCT-116 and Caco-2 control cells to the preG_0_ in cells treated with the IC_50_ of *T. boudieri* water extract, an indication of DNA fragmentation, and a hallmark of apoptosis. The cell count in both the S phase and G_2_-M was diminished. Treated cells showed an evident shift toward the preG_0_ phase where the percentage of the cells in the cells treated with IC_50_ was 60.9% for HCT-116 cells and 54.6% for the Caco-2 cells. The average percentage of each phase is denoted in the figures.

Cell cycle analysis detected considerable DNA fragmentation, a hallmark of apoptosis, in all treated samples. Due to the extensive DNA fragmentation, it was difficult to determine any cell cycle arrest, and the percentage of cells beyond the G_0_-G_1_ phase was greatly diminished. The maximum percentage of cells in the G_2_-M phase was approximately 4% for both HCT-116 and Caco-2 cells exposed to the water extract (Figure 6).

### 3.8. *T. boudieri* Water Extract Downregulated Bcl-2, cyclinD1, and c-Myc, and Upregulated p53 and Bax Proteins in Cancer Cells

**Figure 7:**
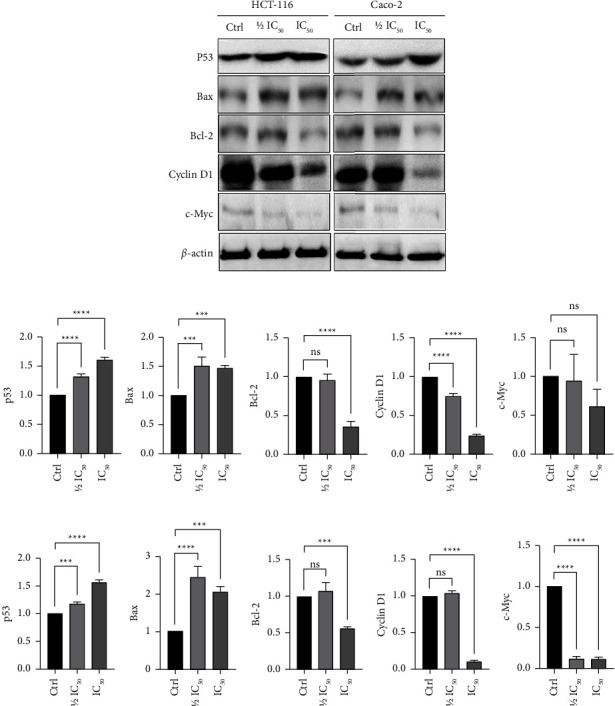
(a) A drop in the protein ratio of cyclin D1, c-Myc, and Bcl-2 in HCT-116 and Caco-2 cells treated with the water extract of *T. boudieri* and a rise in that of p53 and Bax is observed mostly in a dose-dependent manner. Protein levels in HCT-116 cells (b) and Caco-2 cells (c) reflected in the thickness and intensity of the bands in the western blot which varied with treatment was indicative of the effect of the treatment. Band intensity was measured using ImageJ (U.S. National Institutes of Health, Bethesda, Maryland, USA, https://imagej.nih.gov/ij/) and quantification was performed according to the protocol of quantifications of western blots with ImageJ by Hossein Davarinejad [31].

**Figure 8:**
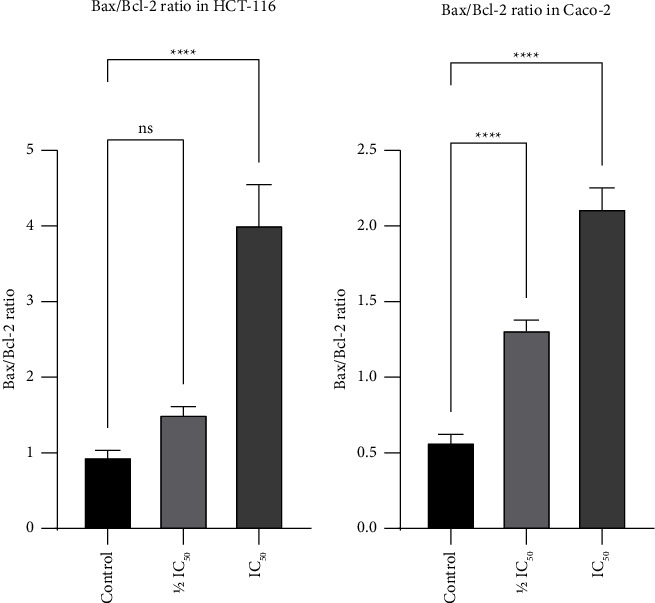
Bax/Bcl-2 ratio in (a) HCT-116 and (b) Caco-2 cells treated with IC50 and 1/2 IC50 of the water extract of *T. boudieri.*

After treatment of HCT-116 and Caco-2 cells with *T. boudieri* water extract, there was a statistically significant change in the expression for all proteins analyzed compared with the negative control. The levels of p53 increased in both treated cancer cell lines in a dose-dependent manner. The levels of Bax were also significantly increased after treatment in both cell lines. In contrast, the levels of Bcl-2 (an inhibitor of Bax) were significantly decreased in both cell lines after treatment with *T. boudieri* water extract (Figure 7). In turn, these changes resulted in a significant rise in the Bax/Bcl-2 ratio after treatment (Figure 8). The treatment also led to a reduction in the levels of cyclin D1 and c-Myc. Cyclin D1 is an important player in cell cycle progression into the S phase, [32] and c-Myc is a master regulator that is highly expressed in many types of cancer leading to an increase in the expression of many genes involved in cellular proliferation [9] (Figure 7).

## 4. Discussion

Several studies have demonstrated that natural extracts have the capacity to induce cytotoxicity in cancer cells while sparing normal cells. This property renders them attractive candidates for the development of therapeutic agents [33–36]. A possible explanation for this difference may be the higher metabolic rates of cancer cells and the consequent increase in the demand for nutrient acquisition, which sensitize them to treatment compared with normal cells [37, 38].

Africa and the Middle East countries have a rich history in the treatment of various ailments using traditional medicines derived from local plants and macrofungi. A poem found in an Egyptian temple provides us with clues regarding the reverence with which the natives beheld macrofungi: “Without leaves, without buds, without flowers: yet they form fruit; as a food, as a tonic, as a medicine: the entire creation is precious [39].” *T. boudieri* is a desert truffle abundantly found in the arid and semiarid areas of the Middle East, and is considered a part of the native cuisine. Studies have investigated its chemical composition and found that it is rich in numerous compounds (e.g., carbohydrates, alkaloids, tannins, flavonoids, steroids, and terpenoids), linoleic acid and glutamic acid, essential amino acids (e.g., leucine and threonine), and elements (e.g., magnesium) [19, 21]. Furthermore, studies have shown that this desert truffle base may have immunomodulatory, antioxidant, and anticancer properties [29]. Al-Obaydi et al. demonstrated that various extracts of *T. boudieri* exert a significantly greater effect against the survival of cancer cells (T47D, MCF-7, MDA-MB231, HCT-116, and Hela) versus kidney epithelial cells (Vero cells) at all extract concentrations examined (i.e., 0.75–25 mg/ml) [21]. However, the mechanisms underlying this influence on cellular physiology remains to be fully understood. In this study, HCT-116 and Caco-2 colon cancer cell lines and western blotting were used to investigate several proteins (i.e., Bax, Bcl-2, p53, CCND1, and c-Myc) that play a central role in cell apoptosis, proliferation, and cell cycle progression.

The cell viability assay revealed that both extracts exerted a comparable cytotoxic exert on the cell lines. For HCT-116 and Caco-2 cells, the IC_50_ of the ethanolic extract was 4.8 mg/ml and 6.4 mg/ml, respectively, while that of the water extract was 4 mg/ml and 5.4 mg/ml, respectively (Figure 1). This indicates that the HCT-116 cell line was more sensitive to treatment than Caco-2 cells. Moreover, the water extract had a slightly greater effect on cells than its ethanolic counterpart. This could be attributed to the higher carbohydrate and phenolic content of the water extract [21]. The crystal violet assay, conducted using the water extract, yielded comparable results. However, the water extract showed lower IC_50_ values for both cell lines (2.65 mg/ml and 3.96 mg/ml for HCT-116 and Caco-2 cells, respectively) than those of the ethanolic extract. These lower values are potentially important because of the use of tap water that could result in stronger detachment of treated cells (Figure 2). HCT-116 cells were more sensitive to the treatment than Caco-2 cells.

In the wound healing assay, the water extract inhibited wound healing at 48 h after treatment, even at the lowest concentrations used (i.e., 1/4 IC_50_). In the wells where the two cell lines (HCT-116 and Caco-2) were treated with their respective IC_50_ of *T. boudieri* water extract, the wound area was greater than that recorded at the time of wound infliction. In addition, few cells were present around the wound and some showed morphological signs of apoptosis under the microscope (e.g., shrinkage and darkening of the nucleus) (Figure 5). This observation was confirmed in the cytomorphological study, in which signs of apoptosis were present in a dose-dependent manner in both the cell lines treated with the water extract (Figure 3). Both these experiments verified that HCT-116 cells are more sensitive to treatment than Caco-2 cells. These findings suggest that the extract has a cytotoxic effect on cancer cells, and affects their migration and proliferation. This was supported by the results of the clonogenic assay (Figure 4), in which cells treated for 48 h showed a significant dose-dependent reduction in their capacity to form colonies compared with the negative control.

Flow cytometry for cell cycle analysis after treatment with IC_50_ and 1/2 IC_50_ of water extract for 48 h showed extensive fragmentation (preG_0_ was approximately 40–65%, with higher percentages observed for cells treated with IC_50_). This is a hallmark of apoptosis that aligns with the findings of the previous experiments. However, given the high percentages of the preG_0_ state, it was difficult to determine the effect of the water extract on the cell cycle. Notably, the highest and lowest percentages of cells were found in the G_0_-G_1_ and G_2_ phases, respectively. Moreover, it was shown that cyclin D1 is a major driver of the cell cycle beyond G_1_ [32]. The western blotting results showed a remarkable decrease in the levels of cyclin D1 in cells treated with the IC_50_ of the water extract (Figure 7). Therefore, it is reasonable to conclude that few treated cells would transition into the S phase.

p53 is involved in a wide range of cellular processes (e.g., differentiation, DNA repair, cellular senescence, cell death, and cell cycle) [40]. Dysregulation of p53 through mutation or deactivation of its signaling capacities is important for cancer development [5]. This suppression of p53, found in up to 43% of cancers and 60% of patients with colorectal cancer, drives the development of cancer and is an important prognostic indicator in this setting [41]. The present results showed a significant increase in the levels of p53 in both colon cancer cell lines in a dose-dependent manner following treatment with the water extract of *T. boudieri* (Figure 7). It has been previously shown that flavonoids present in the aqueous/water extracts of *T. boudieri* [21] induce apoptosis and cell cycle arrest through p53 accumulation [42]. The results of the western blotting analysis revealed a rise in the levels of another protein that plays a central role in the mitochondrial or intrinsic pathway of apoptosis, namely Bax. When activated, Bax enters into the mitochondrial membrane, thereby inducing its permeabilization, the release of cytochrome c into the cellular cytoplasm, and the formation of the apoptosome which leads to the induction of apoptosis [43]. Dysregulation of apoptosis results in the disruption of tissue turnover, and this process is associated with the occurrence of neoplasms [44]. Bcl-2 plays an important role in suppressing apoptosis by inhibiting the binding of Bax to the mitochondrial membrane [43]. The antiapoptotic activity of Bcl-2 confers resistance to chemotherapy in patients with cancer [45]. Therefore, Bcl-2 has been identified as a target for cancer therapy [46, 47]. Moreover, the Bax/Bcl-2 ratio has a clinical significance in the prognosis of patients with cancer and the development of resistance to chemotherapeutic agents [11, 48]. Treatment of HCT-116 and Caco-2 cells with their respective IC_50_ of *T. boudieri* water extract led to a significant decrease in the protein levels of Bcl-2 (Figure 7) and a significant rise in the Bax/Bcl-2 ratio (Figure 8). The canonical Wnt pathway or Wnt/*β* catenin pathway is another important pathway in cancer development. This pathway is commonly overactivated in colon cancer [49–51]. The endpoint of this pathway is the activation of the transcription factor/lymphoid enhancer binding factor (TCF/LEF) family [52]. A characteristic of this activation is the expression of the c-Myc gene, a target of TCF4. c-Myc is a master regulator that can directly repress p21, thereby causing hyperproliferation [53–55].

Increased Wnt signaling leads to overexpression of the CCDN1 gene. cyclin D1 is an important regulator of the cell cycle, promoting the transition from the G1 phase to the S phase [56]. It is also implicated in promoting the invasion and dissemination of cancer cells [32]. Furthermore, a coordinated effect between c-Myc and cyclin D1 may drive tumor aggressiveness and accelerate disease progression [8]. Thus, the roles of c-Myc, cyclin D1, and the Wnt pathway in the treatment of cancer have been extensively studied [50, 57, 58]. Similar to Bax and Bcl-2, c-Myc and cyclin D1 have been suggested as prognostic factors in cancer [59]. In this study, the protein levels of cyclin D1 and c-Myc were significantly decreased in HCT-116 and Caco-2 cells after treatment with their respective IC_50_ of the water extract; this reduction was more notable in Caco-2 cells.

Collectively, the results of this study provide a more in-depth description of the processes underlying the cytotoxic effect of *T. boudieri*. Al-Obaydi et al. [21] previously demonstrated the apoptotic effect of *T. boudieri* extracts through a caspase 3 (CASP3) assay. In addition, Dahham et al. demonstrated the cytotoxic effect of various extracts of *T*. *boudieri* on different cell lines using the MTT cytotoxicity assay, through loss of the mitochondrial membrane potential [29].

## 5. Conclusions

*T. boudieri* water and ethanolic extracts exerted comparable cytotoxic effects on colon cancer cells, HCT-116 and Caco-2. The slightly more potent water extract was able to reduce cancer cell stemness. The treatment reduced the capacity of cells for proliferation and migration. The morphological characteristics of apoptosis were also evident following treatment. Further analysis revealed extensive DNA fragmentation after treatment, a hallmark of apoptosis. The protein levels of p53 and Bax were elevated in treated cells, whereas those of Bcl-2, c-Myc, and CCND1 were reduced. This suggests that *T. boudieri* water extract exerts a proapoptotic effect on colon cancer cells through multiple signaling pathways. In addition, the extract also demonstrated an antiproliferative effect. The extract simultaneously affected the levels of various proteins considered as important targets in cancer therapy. Further investigation is warranted to determine the usefulness of *T. boudieri* and its constitutive elements as an adjunct to current chemotherapy for the treatment of cancer.

## Data Availability

The data generated during and/or analyzed during the current study are available from the corresponding author upon reasonable request.
